# Clinical and genetic markers associated with tuberculosis, HIV-1 infection, and TB/HIV-immune reconstitution inflammatory syndrome outcomes

**DOI:** 10.1186/s12879-020-4786-5

**Published:** 2020-01-20

**Authors:** Nathalia Beatriz Ramos de Sá, Marcelo Ribeiro-Alves, Tatiana Pereira da Silva, Jose Henrique Pilotto, Valeria Cavalcanti Rolla, Carmem B. W. Giacoia-Gripp, Daniel Scott-Algara, Mariza Gonçalves Morgado, Sylvia Lopes Maia Teixeira

**Affiliations:** 1 Laboratory of AIDS & Molecular Immunology, Oswaldo Cruz Institute, FIOCRUZ. Av. Brasil 4365, Leonidas Deane Building, room 401, Rio de Janeiro, 21040-360 Brazil; 2 Laboratory of Clinical Research on STD/AIDS, National Institute of Infectious Diseases Evandro Chagas, FIOCRUZ, Rio de Janeiro, Brazil; 3 Nova Iguaçu General Hospital, Nova Iguaçu, Rio de Janeiro Brazil; 4 Clinical Research Laboratory on Mycobacteria, National Institute of Infectious Diseases Evandro Chagas, FIOCRUZ, Rio de Janeiro, Brazil; 5 Unité de Biologie Cellulaire des Lymphocytes, Institut Pasteur, Paris, France

**Keywords:** Tuberculosis, HIV-1, Immune Reconstitution Inflammatory Syndrome, HLA-B genes, HLA-C genes, KIR genes

## Abstract

**Background:**

Tuberculosis (TB) and AIDS are the leading causes of infectious disease death worldwide. In some TB-HIV co-infected individuals treated for both diseases simultaneously, a pathological inflammatory reaction termed immune reconstitution inflammatory syndrome (IRIS) may occur. The risk factors for IRIS are not fully defined. We investigated the association of HLA-B, HLA-C, and KIR genotypes with TB, HIV-1 infection, and IRIS onset.

**Methods:**

Patients were divided into four groups: Group 1- TB+/HIV+ (*n* = 88; 11 of them with IRIS), Group 2- HIV+ (*n* = 24), Group 3- TB+ (*n* = 24) and Group 4- healthy volunteers (*n* = 26). Patients were followed up at INI/FIOCRUZ and HGNI (Rio de Janeiro/Brazil) from 2006 to 2016. The HLA-B and HLA-C loci were typed using SBT, NGS, and KIR genes by PCR-SSP. Unconditional logistic regression models were performed for Protection/risk estimation.

**Results:**

Among the individuals with TB as the outcome, KIR2DS2 was associated with increased risk for TB onset (aOR = 2.39, *P* = 0.04), whereas HLA-B*08 and female gender were associated with protection against TB onset (aOR = 0.23, *P* = 0.03, and aOR = 0.33, *P* = 0.01, respectively). Not carrying KIR2DL3 (aOR = 0.18, *P* = 0.03) and carrying HLA-C*07 (aOR = 0.32, *P* = 0.04) were associated with protection against TB onset among HIV-infected patients. An increased risk for IRIS onset was associated with having a CD8 count ≤500 cells/mm^3^ (aOR = 18.23, *P* = 0.016); carrying the KIR2DS2 gene (aOR = 27.22, *P* = 0.032), the HLA-B*41 allele (aOR = 68.84, *P* = 0.033), the KIR2DS1 + HLA-C2 pair (aOR = 28.58, *P* = 0.024); and not carrying the KIR2DL3 + HLA-C1/C2 pair (aOR = 43.04, *P* = 0.034), and the KIR2DL1 + HLA-C1/C2 pair (aOR = 43.04, *P* = 0.034),

**Conclusions:**

These results suggest the participation of these genes in the immunopathogenic mechanisms related to the conditions studied. This is the first study demonstrating an association of HLA-B*41, KIR2DS2, and KIR + HLA-C pairs with IRIS onset among TB-HIV co-infected individuals.

## Background

Approximately 36.9 million people worldwide were living with HIV infection in 2017, and 6.3 million new cases of tuberculosis (TB) were reported in 2016 [1]. Of these 6.3 million people with TB, 7.6% (476,774) were also HIV-1 infected, of whom 85% were on combined antiretroviral therapy (cART), making TB the most common opportunistic infection leading to death among HIV-1 patients [1]. Approximately 69,500 new cases of TB were reported in 2016 in Brazil, and 9.4% of these cases were associated with HIV-1 infection [2]. In Rio de Janeiro, the Brazilian state where the individuals included in this study were recruited, the incidence rate of TB was 61.2/100,000 inhabitants in 2016, 8.9% of which also had an associated HIV-1 infection [2].

Genetic studies have provided valuable insights into the resistance, susceptibility, and progression of infectious diseases since the enormous diversity of phenotypes associated with these diseases reflects the heterogeneous composition of host genotypes. Studies have suggested that both innate and adaptive immunity are involved in the pathogenesis of infectious diseases [3, 4]. Therefore, the characterization of immune response genes is an important step in understanding the factors that can lead to TB and/or HIV-1, TB-HIV co-infected individuals, and IRIS onset. It is known that HLA (human leukocyte antigen) class I and KIR (killer-cell immunoglobulin-like receptor) genes influence the outcomes of HIV-1 infections and TB [5–7]. In particular, the HLA-B locus plays a dominant role in the selection of cytotoxic T-lymphocyte (CTL) responses when compared with other class I molecules [7–9]. HLA-C has a dual role of presenting antigens to CTL and serving as ligands to KIR receptors on NK cells, thus regulating the lysis of target cells mediated by NK cells [10]. Many KIR genes and KIR-HLA-B/KIR-HLA-C pairs have been associated with distinct outcomes in the context of HIV-1 infection [6, 11–14]. In the same way, genetic studies involving protection or susceptibility to pulmonary tuberculosis have also highlighted the role of HLA-B and KIR genes, as well as KIR + HLA-C pairs [5, 15–18].

On the other hand, studies associating both innate and adaptive immune response genes with outcomes of TB-HIV co-infection are scarce. Some of the markers already described are HLA-A, −B, and -DRB1 alleles [19, 20]. The management of TB-HIV co-infected individuals may have specific characteristics that can bring complexity to its dynamics. One example is the improvement of survival provided by the use of cART during TB treatment, which can restore immune function [21]. However, simultaneous treatment with anti-TB drugs and cART can lead to a paradoxical clinical worsening with exacerbation of the immune response, known as immune reconstitution inflammatory syndrome (IRIS) [21, 22]. The syndrome is currently classified into two forms, named paradoxical IRIS and unmasked IRIS. In paradoxical IRIS, the signs and symptoms of a pre-existing opportunistic infection, partially treated, recur, or worsen intensely despite a positive response before cART [23, 24]. Unmasked IRIS is characterized by the discovery of a previously undiagnosed/latent infection. In this way, the signs and symptoms of opportunistic infection do not appear initially, appearing after the introduction of cART [23, 24]. IRIS has been associated with a large variety of other pathogens and autoimmune diseases [21, 25, 26], but mycobacterial infections are the most prevalent cause of IRIS [21, 27, 28]. TB/HIV-IRIS occurs in 4–54% of patients starting cART during TB treatment [29], depending on various features. In South America, this estimated incidence was 10% in an extensive meta-analysis comparing TB/HIV-IRIS cases from different parts of the world [30]. The few Brazilian studies regarding IRIS report an estimated incidence of approximately 12%, which limits the recruitment and analysis of these individuals [31, 32]. Pathogenic mechanisms involved in IRIS development have been suggested, such as antigenic load, degree of immune restoration after treatment with cART, and genetic susceptibility of the host, and there is evidence that these mechanisms can interact with each other and together cause the syndrome [23, 25, 33]. However, studies linking host genetics to the pathogenesis of IRIS are still scarce [34–37]. Based on the microarray analysis of gene expression of isolated monocytes, Tran et al. (2013,2014) [34, 35] demonstrated upregulation of genes related to the role of pathogen pattern recognition to bacteria and viruses and the complement system, highlighting the potential role of monocytes and complement in the predisposition/development of TB-IRIS. Besides, Affandi et al. (2013) [36] demonstrated that the susceptibility to TB-IRIS was associated with the presence of specific single nucleotide polymorphisms (SNPs) of cytokine-related genes.

Moreover, clinical risk factors already known to be associated with IRIS pathogenesis are (a) low baseline CD4^+^ T-cell count (< 50–100 cells per mm^3^) combined with a short time interval between the beginning of TB treatment and cART [38–42] and (b) dissemination of TB to extrapulmonary sites, possibly reflecting a high bacterial load [43, 44]. Nevertheless, despite the few biomarker descriptions associated with IRIS, there is still no one capable of predicting IRIS development currently being used in the clinical practice.

Innate and adaptive immunity are directly involved in the pathogenesis of IRIS [3, 26, 44, 45]. Characterization of immune response genes is an important approach to assess genetic profiles that could be associated with susceptibility/resistance to the syndrome. In this way, this study aimed to investigate the distribution of HLA-B, HLA-C, and KIR genotypes and their potential influence on susceptibility and/or resistance to TB and/or HIV-1 as well as on the occurrence of TB-IRIS.

## Methods

### Patients’ enrolment and study design

This is a genetic study nested in two clinical and immunological follow-up studies previously conducted in the Laboratory of AIDS and Molecular Immunology (IOC/FIOCRUZ), which assessed immunological characteristics of TB-HIV co-infected individuals and the risk factors for paradoxical TB/HIV-IRIS [32, 46, 47]. The HLA-B, HLA-C and KIR genetic profiles were determined from 162 individuals divided into four groups as follows: Group 1 - individuals infected with HIV-1 and tuberculosis (HIV+/TB+ group, *n* = 88; 11 of them with IRIS); Group 2 - individuals infected with HIV-1 without diagnosis of TB (HIV-1+ group, *n* = 24); Group 3 - individuals with tuberculosis and seronegative for HIV-1 infection (TB+ group, *n* = 24); and Group 4 - healthy volunteers without HIV-1 infection and/or TB (control group, *n* = 26).

Individuals were enrolled and followed up at the Tuberculosis Clinic of the National Institute of Infectious Diseases Evandro Chagas, Oswaldo Cruz Foundation (INI/FIOCRUZ), Rio de Janeiro, Brazil from 2006 to 2011 [32, 46] and at the Nova Iguaçu General Hospital (HGNI), Rio de Janeiro, Brazil from 2014 to 2016. For this study, recruited patients were eligible if they were 18 years old or older. Group 1 included individuals with TB newly diagnosed for HIV-1 infection, with CD4+ T-cell count < 350 cells/mm^3^, starting consecutive (30 ± 10 days interval) anti-tuberculosis and cART treatments. The exclusion criteria were as follows: (1) for Group 1 and Group 2 - baseline hepatic enzymes elevation, CD4+ T-cell count above 350 cells/mm^3^ at the time of tuberculosis diagnosis, and being on antiretroviral and/or anti-tuberculosis treatment and developing tuberculosis, to exclude unmasking IRIS; (2) for Group 3 - being on treatment for tuberculosis; and (3) for Group 4 - could not be diagnosed with HIV, TB, hepatitis and other diseases. Patients included in groups 1 to 3 were starting cART and/or anti-tuberculosis treatments, respectively, prescribed according to the Brazilian Ministry of Health guidelines [48] and the National Tuberculosis Program [49]. cART therapy was offered according to contemporary Brazilian National Guidelines that were periodically updated [48] using two nucleoside reverse transcriptase inhibitors (NRTI) + one non-nucleoside reverse transcriptase inhibitor (NNRTI). The anti-tuberculosis treatment was composed of the combination of rifampicin, isoniazid, pyrazinamide, and, from 2009 on, ethambutol, according to the recommendation of the National TB program of the Brazilian Ministry of Health [49].

During follow-up, Group 1 patients were investigated for the identification of IRIS development in both clinical centers. All IRIS cases observed in the study were classified as paradoxical, tuberculosis-associated IRIS, described as an worsening of TB signs and symptoms starting after cART initiation during TB-treatment, mainly presenting enlargement of lymph nodes and inflammatory signs, not explained by any other diseases or by an adverse effect of drug therapy [50, 51], as recently detailed/reviewed by our group [52]. In general, the IRIS cases included in the present study were self-resolving, or, if necessary, the patients were treated with corticoid-based therapy, such as Prednisone.

Demographic and clinical data throughout the follow-up period, as well as blood samples from the baseline visit, were available for the present study. Skin color was self-declared.

### Genomic DNA extraction

DNA was extracted from whole blood using the QIAamp DNA Blood Mini Kit (Qiagen, Hilden, Nordrhein-Westfalen, Germany) according to the manufacturer’s instructions. The DNA concentration was determined using a Thermo Scientific NanoDrop 2000 (Thermo Fisher Scientific, Waltham, Massachusetts, USA), and the filtrates containing the isolated DNA were stored at −20 °C until the genomic analyses.

### HLA typing

High-resolution HLA typing of HLA-B and HLA-C genes was determined by sequencing-based typing (SBT) according to the manufacturer’s instructions on an ABI platform using commercial kits (SeCore® Sequencing Kit – Invitrogen by Life Technologies, Brown Deer, Wisconsin, USA). HLA-B and HLA-C genes were assigned using a four-digit designation using uTYPE® v6.0 SBT software (Invitrogen by Life Technologies, Brown Deer, Wisconsin, USA), which also solves the ambiguous results. Due to technical issues, HLA-C typing was performed for 21 individuals by next-generation sequencing (NGS) - MiSeq platform. A pre-validation assay showed that the alleles assigned by SBT and by NGS are the same, which means that the results are comparable (data not shown). The grouping of HLA-B genes in HLA Bw4 and/or Bw6 epitope-associated specificities followed the Immuno Polymorphism Database (IPD)-international ImMunoGeneTics project (IMGT)/HLA nomenclature guidelines [53]. All HLA-B alleles with the Bw4 epitope were grouped, regardless of the amino acid composition in the position 80 (80I or 80 T). In this study, we did not evaluate HLA-Bw4 epitope-associated specificity found in some HLA-A alleles. The grouping of HLA-C genes in C1 (HLA-C*01/*03/*07/*08/*12/*14/*16) and C2 (HLA-C*02/*04/*05/*06/*15/*17/*18) epitope-associated specificities was based in the classification broadly used in the literature [54, 55].

### KIR genotyping

The presence or absence of KIR genes was determined using a commercial kit based on a sequence-specific primer amplification method – SSP (SSP KIR Genotyping kit – Invitrogen, Brown Deer, Wisconsin, USA). A total of 14 KIR genes (2DL1, 2DL2, 2DL3, 2DL4, 2DL5, 2DS1, 2DS2, 2DS3, 2DS4, 2DS5, 3DL1, 3DL2, 3DL3, and 3DS1) and 2 KIR pseudogenes (2DP1 and 3DP1) were screened using this approach. KIR group genotype nomenclature was designated according to the current working definition, which characterizes genotypes AA and Bx based on the combinations of haplotype A (absence of all the activating genes, except KIR2DS4) and haplotype B (presence of one or more of the activating genes) [12, 56]. We also classified KIR genotypes by the ID assigned by the Allele Frequency Net Database (http://www.allelefrequencies.net). KIR-HLA pairs were named according to the presence of at least one allele of a particular allotype (Bw4 or Bw6; C1 or C2), as follows: Bw4/Bw4, Bw4/Bw6, Bw6/Bw6 and C1/C1, C1/C2, C2/C2.

### Statistical analyses

Mann–Whitney U tests were used in the comparisons of the sociodemographic, clinical, and laboratory characteristics for continuous numerical variables, while for categorical nominal variables, Fisher’s exact tests were used in the evaluation of frequencies between groups. For the last, *P*-values were computed by Monte Carlo simulation with B = 1,000,000 simulations [57]. The frequencies of HLA-B, HLA-C, and KIR genes and genotypes were determined by direct count, and their proportions within 95% confidence intervals (CI) were computed according to the Gamma distribution [58]. The protection/risk estimation was performed using an adjusted odds ratio (aOR) with 95% CI for each gene and estimated through unconditional logistic regression models. We introduced as confounders, any clinical phenotypic marker associated with the different outcomes in the modeling of all other genetic or phenotypic analysis to eliminate any possible bias introduced for having more or fewer individuals living with HIV in the aOR numerator or denominator of any analysis. Whenever needed, we categorized continuous numerical variables using as cut-offs the round integer number closest to the medians of the outcome’s sets defined by the continuous numeric variable. Sociodemographic, clinical, and laboratory characteristics with the outcomes of interest and *P*-values <0.2 in the bivariate analysis were included in multiple unconditional logistic regression models to account for biases. All statistical analyses were performed using R version 3.6.0 (R Core Team, 2019). HLA allele frequencies were also compared between the different groups of patients and individuals from the Brazilian National Registry of Bone Marrow Donors (REDOME) released in March 2013, which represents a reliable and representative sample of the Brazilian population, with almost 3 million registered donors (www.imunogenetica.org).

## Results

### Clinical and epidemiological characteristics

The main sociodemographic, clinical, and laboratory features of all individuals included in the present study, categorized according to the presence (Group 1 + Group 3) or absence (Group 2 + Group 4) of TB, are depicted in Table 1. Among the 88 TB-HIV co-infected individuals included in the study, 11 had paradoxical TB/HIV-IRIS. Most of the participants were males (69.8%). Regarding schooling, 41.4% of the individuals have lower secondary education, and 30.2% have upper secondary education. The proportion of individuals with white and brown skin color was equivalent (40.7 and 39.5%, respectively), and individuals with black skin color made up the remainder of the study population (19.8%). The education level, gender, HIV status, CD4 T-cell count/mm^3,^ and CD4/CD8 ratio were significantly distinct between the groups of individuals with (Group 1 + Group 3) and without (Group 2 + Group 4) TB (Table 1).

**Table 1:** Sociodemographic, clinical, and laboratory data for individuals included in the study categorized according to the presence (Group 1 + Group 3) or absence (Group 2 + Group 4) of TB

Features	Overall *N* = 162	With TB (G1 + G3) *N* = 112	Without TB (G2 + G4) *N* = 50	*P-*value^a^
Gender; n (%)				
Female	49 (30.2)	26 (23.21)	23 (46)	**0.005**
Male	113 (69.8)	86 (76.79)	27 (54)	
Skin Color^b^; n (%)				
Black	32 (19.8)	25 (22.32)	7 (14)	0.379
Brown	64 (39.5)	41 (36.61)	23 (46)	
White	66 (40.7)	46 (41.07)	20 (40)	
Education^c^; n (%)				
Bachelor	13 (8)	6 (5.36)	7 (14)	**<0.001**
Upper-secondary	49 (30.2)	27 (24.11)	22 (44)	
Lower-secondary	67 (41.4)	46 (41.07)	21 (42)	
Primary	26 (16)	26 (23.21)	0 (0)	
Unknown	7 (4.3)	7 (6.25)	0 (0)	
HIV status; n (%)				
Negative	50 (30.9)	24 (21.43)	26 (52)	**0.0002**
Positive	112 (69.1)	88 (78.57)	24 (48)	
CD4 count (cell/μL) (IQR)	153 (654.25)	130.5 (116.51)	674 (1348)	**0.003**
(≤50); n (%)	40 (25.3)	28 (25)	12 (24)	0.846
(>50); n (%)	118 (74.7)	80 (71.43)	38 (76)	
CD8 (IQR)	588 (562)	591 (527.7)	564 (1.128)	
(≤500); n (%)	65 (42.5)	44 (39.28)	21 (42)	1
(>500); n (%)	88 (57.5)	59 (52.68)	29 (58)	
CD4/CD8 (IQR)	0.29 (0.96)	0.2 (0.18)	0.69 (1.38)	**0.004**
(≤1); n (%)	111 (72.5)	85 (75.89)	26 (52)	**0.0002**
(>1); n (%)	42 (27.5)	18 (16.07)	24 (48)	

*N* number of individuals in each group, *TB* tuberculosis, *IQR* interquartile range, *VL* viral load, *G1* group 1, *G2* group 2, *G3* group 3, *G4* group 4

^a^*P*-values were calculated using Fisher’s exact test. Differences were considered significant with a value of * *P* < 0.05. Significant *P*-values are labeled in bold. ^b^Skin color categorization followed the classificatory system employed by the Brazilian Institute of Geography and Statistics (IBGE) [59]. ^c^Classification, according to the International Standard Classification of Education (ISCED) maintained by the United Nations Educational, Scientific and Cultural Organization (UNESCO)

We further analyzed the main sociodemographic, clinical, and laboratory characteristics of HIV-1-infected individuals included in this study, also categorized according to the presence (Group 1) or absence (Group 2) of TB, as depicted in Additional file 1: Table S1. A total of 75% of HIV-1-infected individuals identified themselves as heterosexual and 23.2% as men who have sex with men (MSM). Most males (62.5%) and heterosexual subjects (58.9%) belonged to Group 1. The education level was diverse, with an unequal distribution between Group 1 and Group 2 (*P* = 0.011). There were no statistically significant differences in clinical and laboratory variables between Group 1 and Group 2 patients (Additional file 1: Table S1).

### Distribution of HLA-B, HLA-C, and KIR genes

All 162 individuals had their HLA-B genotypes determined, and 30 allelic groups were identified. Given the large variety of the specific HLA-B alleles detected, we opted to present the results using the two-digit designation, in an attempt to facilitate the interpretation of the associations here described. The most frequent HLA-B alleles were HLA-B*15, HLA-B*44, HLA-B*35 and HLA-B*07, with prevalence rates of 26.5, 20.4, 18.5, and 14.2%, respectively (Additional file 1: Table S2). Twenty-four individuals (14.8%) were HLA-Bw4 homozygous, and 53 (32.7%) were HLA-Bw6 homozygous, while Bw4/Bw6 heterozygosity was found in 85 individuals (52.5%) (Additional file 1: Table S2). No statistically significant differences were observed between the HLA-B serological epitopes and the groups studied (Additional file 1: Table S3). Comparing the frequencies of all HLA-B alleles found in this study with those reported for the general Brazilian population (REDOME dataset), we observed a significant difference in the frequencies of HLA-B*15, HLA-B*53, HLA- B*81 alleles (Additional file 1: Table S4).

One hundred sixty out of the 162 individuals included in the study had their HLA-C genotypes determined, and 14 allelic groups were identified. The DNA samples from the remaining two individuals were submitted repeatedly times to the sequencing protocol, but the typing was unsuccessful. A large variety of specific HLA-C alleles was also detected, and, as well as was done for HLA-B alleles, and we opted to present the results using the two-digit designation. The most frequent HLA-C alleles were HLA-C*07, HLA-C*04, HLA-C*06, and HLA-C*03, with prevalence rates of 40.0, 37.5, 19.4, and 18.8%, respectively (Additional file 1: Table S2). Forty-three individuals (26.9%) were HLA-C1 homozygous, and 41 individuals (25.6%) were HLA-C2 homozygous, while seventy-six individuals (47.5%) were HLA-C1/C2 heterozygous (Additional file 1: Table S2). Unfortunately, HLA-C frequencies are not available at the REDOME dataset for comparisons of our data with the general Brazilian population.

All 16 KIR genes (14 genes and 2 pseudogenes) were detected in the study population. One hundred sixty-one out of the 162 individuals included in the study had their KIR genotypes determined. These profiles ranged in their frequency from 0.62% (1/161) to 28.6% (46/161). Twenty-two of the 43 profiles identified were unique to a single individual, reinforcing the high degree of polymorphism of these genes. The most representative KIR genotypes were AA1 (46/161, 28.6%), Bx4 (16/161, 9.9%), Bx5, Bx6 (11/161, 6.8%), and Bx2 (10/161, 6.2%) (Additional file 1: Table S5). The inhibitory KIR genes were more frequent than the activating genes (58.7% vs. 24.5%) (Additional file 1: Tables S2 and S5). Considering the AA and Bx genotype classification, 112 individuals (69.1%) had KIR genes arranged as Bx haplotypes, and in the 50 remaining individuals (30.9%), the AA genotype was observed (Additional file 1: Table S2). The KIR Bx genotype was more frequent among all groups of patients, with 70.4% in Group 1, 66.7% in Group 2, 75.0% in Group 3, and 61.5% in Group 4 (Additional file 1: Table S2). Statistically significant differences were not observed between the HLA-B serological epitopes and the groups studied (Additional file 1: Table S6).

The results of the unconditional logistic multiple regression model comparing the groups with TB (Group 1 + Group 3) and without TB (Group 2 + Group 4) showed that HLA-B*08 [aOR = 0.23 (95% CI, 0.06–0.89), *P* = 0.033] and female gender [aOR = 0.33 (95% CI, 0.13–0.8), *P* = 0.014] were associated with protection against TB onset, while KIR2DS2 was associated with increased risk for TB onset [aOR = 2.39 (95% CI, 1.03–5.54), *P* = 0.043]. Among the HIV-1 infected individuals (Group 1 vs Group 2), not carrying KIR2DL3 [aOR = 0.18 (95% CI, 0.04–0.74), *P* = 0.034] and carrying HLA-C*07 ([aOR = 0.32 (95% CI, 0.11–0.94), *P* = 0.038] were associated with protection against TB onset (Table 2).

**Table 2:** Unconditional logistic multiple regression model of risk and protection factors for tuberculosis

Features	Level	All the groups		G1 + G3 vs. G2 + G4			HIV-1 positive individuals		G1 vs. G2		
		With TB (G1 + G3) *N* = 112	Without TB (G2 + G4) *N* = 50	aOR^a^	95%CI	*P*-value^b^	With TB (G1) *N* = 88	Without TB (G2) *N* = 24	aOR	95%CI	*P*-value
Gender	Male	86 (76.79)	27 (54)	Ref			70 (79.55)	16 (66.67)	Ref		
	Female	26 (23.21)	23 (46)	0.33	0.13–0.8	**0.014**	18 (20.45)	8 (33.33)	0.49	0.17–1.43	0.192
HLA-B*08	not carriers	105 (93.75)	41 (82)	Ref			82 (93.18)	21 (87.5)	Ref		
	carriers	7 (6.25)	9 (18)	0.23	0.06–089	**0.033**	6 (6.82)	3 (12.5)	0.53	0.1–2.77	0.450
KIR2DL3	carriers	105 (93.75)	44 (88)	Ref			84 (95.45)	19 (79.17)	Ref		
	not carriers	7 (6.25)	6 (12)	0.52	0.14–1.89	0.319	4 (4.55)	5 (20.83)	0.18	0.04–0.74	**0.034**
KIR2DS2	not carriers	50 (44.64)	29 (58)	Ref			42 (47.73)	14 (58.33)	Ref		
	carriers	62 (55.36)	21 (42)	2.39	1.03–5.54	**0.043**	46 (52.27)	10 (41.67)	1.74	0.66–4.64	0.265
HLA-C*07^c^	carriers	37 (33.64)^c^	20 (40)	0.75	0.32–1.71	0.489	28 (32.56)^c^	12 (50)	0.32	0.11–0.94	**0.038**
	not carriers	73 (66.36)^c^	30 (60)	Ref			58 (67.44)^c^	12 (50)	Ref		

^a^Odds ratios were adjusted by gender, skin color, education, HIV status, CD4 count, and CD4/CD8 ratio when appropriate. ^b^*P*-values were calculated using the unconditional logistic regression model. Differences were considered significant with a value of ^*^ *P* < 0.05. Significant *P*-values are labeled in bold. ^c^The HLA-C determination was not possible for two individuals from G1. So, when considering this variable, N (G1) = 86 and N (G1 + G3) = 110

*N* number of individuals in each group, *aOR* adjusted odds ratio, *95% CI* 95% confidence interval, *REF* Reference, *G1* group 1, *G2* group 2, *G3* group 3. *G4* group 4

Additionally, according to the presence (Group 1 + Group 3) or absence (Group 2 + Group 4) of TB, a tendency for an association of KIR2DL2 with increased risk for TB onset was observed [aOR = 2.13 (95% CI, 0.93–4.9), *P* = 0.075]. Among the HIV-1-infected individuals (Group 1 vs. Group 2), white skin color was associated with increased risk for TB onset [aOR = 2.62 (95% CI, 0.85–8.09), *P* = 0.092].

### IRIS and genetic markers

Comparing the IRIS vs non-IRIS subgroups, among the TB-HIV co-infected individuals (Group 1), an increased risk for IRIS onset was associated with having a CD8 count ≤500 cells/mm^3^ [aOR = 18.23 (95% CI, 1.71–193.79), *P* = 0.016]; carrying the KIR2DS2 gene [aOR = 27.22 (95% CI, 1.33–558.6), *P* = 0.032], the HLA-B*41 allele [aOR = 68.84 (95% CI, 1.41–3369.9) *P* = 0.033], the KIR2DS1 + HLA-C2 pair [aOR = 28.58 (95% CI, 1.54–530.65) *P* = 0.024]; and not carrying the KIR2DL3 + HLA-C1/C2 pair [aOR = 43.04 (95% CI, 1.32–1404.01) *P* = 0.034], and the KIR2DL1 + HLA-C1/C2 pair [aOR = 43.04 (95% CI, 1.32–1404.01) *P* = 0.034] (Table 3). Additionally, a trend for association with increased risk for IRIS onset was observed for the occurrence of KIR2DS5 [aOR = 5.77 (95% CI, 0.83–39.96), *P* = 0.076], HLA-B*45 [aOR = 45.93 (95% CI, 0.61–3471.86), *P* = 0.083], and disseminated/extrapulmonary TB [aOR = 5.65 (95% CI, 0.79–40.47), *P* = 0.085].

**Table 3:** Unconditional logistic multiple regression model of risk factors for IRIS-TB among HIV-TB individuals

Features	Level	Patients without IRIS (*N* = 77)	Patients with IRIS (*N* = 11)	Adjusted Model		
				aOR^a^	95%CI	*P*-value^b^
CD8	(>500)	47 (68.12)	3 (30)	Ref		
	(≤500)	22 (31.88)	7 (70)	18.23	1.71–193.79	0.016
HLA-B*41	not carriers	75 (97.4)	10 (90.91)	Ref		
	carriers	2 (2.6)	1 (9.09)	68.84	1.41–3369.9	0.033
KIR2DS2	not carriers	40 (51.95)	2 (18.18)	Ref		
	carriers	37 (48.05)	9 (81.82)	27.22	1.33–558.6	0.032
KIR2DS1 + C2	not carriers	69 (89.61)	8 (72.73)	Ref		
	carriers	8 (10.39)	3 (27.27)	28.58	1.54–530.65	0.024
KIR2DL3 + C1/C2	not carriers	37 (49.33)	9 (81.82)	43.04	1.32–1404.01	0.034
	carriers	38 (50.67)	2 (18.18)	Ref		
KIR2DL1 + C1/C2	not carriers	37 (49.33)	9 (81.82)	43.04	1.32–1404.01	0.034
	carriers	38 (50.67)	2 (18.18)	Ref		

*N* number of individuals in each group, *OR* odds ratio, *aOR* adjusted odds ratio, *95% CI* 95% confidence interval, *REF* Reference, *HLA* human leukocyte antigen, *IRIS* immune reconstitution inflammatory syndrome

^a^Odds ratios were adjusted by skin color, education, site of tuberculosis, and CD8 count when appropriate. ^b^P-values were calculated using the unconditional logistic regression model. Differences were considered significant with a value of ^*^ *P* < 0.05

We also observed an unequal distribution of the HIV transmission route (*P* = 0.027) and CD8 count (*P* = 0.032) among the IRIS and non-IRIS groups (Additional file 1: Table S7). Analyses of Bw4/Bw6 epitope groups and AA/Bx genotypes between groups with and without IRIS did not reach statistical significance, contrary to what was observed for the C1/C2 epitope groups (Additional file 1: Table S3 and Additional file 1: Table S6).

## Discussion

The growing interest in the role of host genetic factors in the dynamics of infectious diseases is at least in part fueled by the possibility of finding predictive biomarkers of disease outcomes, such as the occurrence of IRIS in TB-HIV co-infected individuals, contributing to improving clinical management in an attempt to avoid severe disease complications. Several reports have associated polymorphic genes with infectious diseases in different populations and ethnic groups [5, 7, 12, 60, 61]. Host genetic factors have been consistently linked to variations in both susceptibility and resistance to HIV-1 infection and TB [7, 15, 62, 63].

Regarding TB-HIV co-infected individuals, there are few host factors associated with protection or susceptibility mechanisms. The immunological mechanisms underlying the development of IRIS are not yet clearly understood [4, 37, 64, 65]. However, some authors have described potential biomarkers as predictors of IRIS development, for instance, interleukin-18 (IL-18) [4], CXCL10, and IFN-α2 [65]. Similarly, Conesa-Botella et al. reported that tumor necrosis factor (TNF), interferon-gamma (IFN-γ), IL-6, and IL-18 were significantly higher in patients with IRIS [66]. Increased frequencies of IFN-γ-producing cells by Elispot in response to PPD and 38 kDa/CFP-10 antigens were also observed for IRIS patients in a previous study by our group [46]. Concerning natural killer (NK) cells, Pean et al. showed that patients with IRIS had a higher proportion of NK cells degranulation levels of these cells were predictive markers of IRIS development among Cambodian TB-HIV co-infected individuals [3]. Our group performed a similar analysis for a subset of patients here included, but no difference was observed in NK degranulation between IRIS and non-IRIS groups [47]. Also, other groups reported elevated frequencies of KIR-γδ T-cells [67] and CD69+ NK cells [68] in TB-IRIS patients during pre-ART, suggesting that these cells may play a role in IRIS-associated pathology. However, it is not completely elucidated which of these potential biomarkers might have clinical application in predicting IRIS.

In the present study, we examined the distribution of HLA-B, HLA-C, and KIR genes in TB and/or HIV-infected patients and investigated the putative role of these genes in the occurrence of TB/HIV-IRIS. The individuals included in the present study had their HLA-B, HLA-C, and KIR genes determined, and the frequency data observed corroborated what has been described for the HIV-1-infected population [12, 69] and the general Brazilian population [70–75]. HLA-B*08 and female gender were associated with protection against TB onset in the studied population. On the other hand, the KIR2DS2 gene was associated with an increased risk for TB onset (Table 2). To the best of our knowledge, HLA-B*08 has not yet been associated with TB protection. However, an increased frequency of this allele was described among TB-HIV coinfected individuals and HIV-1 infected patients with rapid disease progression, reflecting different roles for this allele in the context of TB and HIV-1 infection [76–79]. Many studies have established links between sex-specific factors and the differential susceptibility or protection to some infectious diseases [80, 81]. TB rates are significantly higher in men than in women [1]. Herzmann and collaborators observed a higher frequency of active TB among men, which could lead to an increased risk for disease progression [82]. KIR2DS2 recognizes HLA-C molecules of the C1 group [83]; there is no previous report associating KIR2DS2 with TB susceptibility. Instead, KIR2DS2 has been associated with rapid disease progression and robust immune activation, accelerating the progress to AIDS [84, 85], and promoting a higher risk to acute lymphoblastic leukemia [86].

Not carrying KIR2DL3 and the carriage of HLA-C*07 were protective factors for TB onset among HIV-1-infected individuals studied here (Table 2). KIR2DL3 recognizes HLA-C molecules of the C1 group [83]. Previous studies have shown that different levels of susceptibility to *M. tuberculosis* may be due to variations in KIR receptors and, consequently, in the repertoire of NK cells [87–89]. In the context of TB, a higher prevalence of KIR2DL3 among TB patients has been observed in several studies [15, 18, 90, 91]. Biberg-Salum et al. [92] showed that HLA-C∗07 allele conferred protection against the development of cytomegalovirus retinitis in Brazilian AIDS patients.

It is noteworthy that all patients who developed TB/HIV-IRIS in our analyses were males. The predominance of males among IRIS patients had already been documented in other studies, but in most of them, there was no association with increased risk of IRIS onset [4, 38, 93]. However, an increased risk of being diagnosed with IRIS was reported for men [93]. We could not confirm this association, given the lack of women with IRIS in our study, which prevented the inclusion of the gender variable in the statistical models.

Interestingly, an increased risk for IRIS onset among TB-HIV co-infected individuals was found among those having a CD8 count ≤500 cells/mm^3^; carrying the KIR2DS2, the HLA-B*41, and the KIR2DS1 + HLA-C2 pair; as well as not carrying KIR2DL3 + HLA-C1/C2 and KIR2DL1 + HLA-C1/C2 pairs (Table 3). HLA-B*41 allotypes have already been associated with susceptibility to TB in patients with AIDS from the northeast region of the state of São Paulo [20], but no association with IRIS has been described for this allele yet. The frequency of the HLA-B*41 allele is low in different populations (Allele Frequency Net Database), differing from the frequency found in the IRIS cases included in the present study. The KIR2DS2 gene was also associated with IRIS onset among TB-HIV co-infected individuals in the present study. The high frequency of this gene described across all studied groups (51.2%) was similar to those observed in several other populations, such as on the African continent (> 54%) and in the Cambodian population (49.9%) [90], where the occurrence of IRIS is higher than that observed in this study [31].

The results regarding activating KIR receptors (KIR2DS2, KIR2DS1 + HLA-C2, and KIR2DS5) together with the lack of inhibitory KIR receptors (KIR2DL3 + HLA-C1/C2 and KIR2DL1 + HLA-C1/C2) might reflect a high functionality of NK cells, suggesting that the presence of these activating genes modulates the NK cell response. This mechanism may be either by no recognition of the activating genes of the infected cells, due to lack of ligands in the target cell, or due to overriding of the activation signal by the inhibitory signal delivered to NK cells when both activating and inhibitory genes bind to their ligand on the surface of the target cell [94–96]. Therefore, this might lead to an escape from the infected cells, resulting in the exacerbation of the pathogenesis of IRIS or HIV-1 infection and TB itself. Future studies should address the functional characterization of these genes and their respective HLA ligands.

To the best of our knowledge, this is the first study showing the scenario of HLA-B, HLA-C, and KIR gene frequencies in a population of HIV-1-infected patients with TB. Importantly, the frequencies of these genes between individuals with and without IRIS were also determined. Our results suggest the participation of the clinical and genetic markers, which were associated with the related TB-HIV outcomes in the immunopathogenic mechanisms related to the conditions studied here. It is relevant to point out that some limitations of the current study should be noted, mainly concerning the limited sample size and the low frequency of TB/HIV-IRIS cases. Therefore, additional studies with larger populations and suitable power analyses might be helpful to a better understanding of the importance and role of genetic host markers in the context of TB and/or TB/HIV-IRIS.

## Conclusions

We conclude that there is a relationship between KIR, HLA-B, and HLA-C genes and the immunopathogenic mechanisms related to the clinical conditions studied here. This one is the first study demonstrating significant associations of the HLA-B*41 allele, the KIR activating receptor gene KIR2DS2, and a combination of KIR/HLA-C pairs with increased risk of IRIS onset among TB-HIV co-infected individuals.

## Supplementary information

**Additional file 1: Table S1.** Sociodemographic, clinical, and laboratory data of HIV-1-positive individuals included in the study categorized according to the presence (G1) or absence (G2) of TB. **Table S2.** Distribution of HLA-B, HLA-C, and KIR genetic profiles found in this study. **Table S3.** Distribution of HLA-B and HLA-C serological epitopes of subjects included in this study stratified by groups. **Table S4.** Distribution of HLA-B alleles of the subjects included in this study and the Brazilian general population (data from the Brazilian Registry of Bone Marrow Donors - REDOME). **Table S5.** Frequency of KIR genotypes and mapping of the inhibitory/activator genes among the subjects included in this study. **Table S6.** Distribution of KIR genotypes of subjects included in this study stratified by groups. **Table S7.** Sociodemographic, clinical, and laboratory data of HIV-TB individuals with and without IRIS.

## Data Availability

All data generated or analyzed during this study are included in the core section and the supplementary information of the additional files. Any additional information will be made available from the corresponding author on a reasonable request.

## Abbreviations

AIDS: Acquired immune deficiency syndrome
cART: Combination anti-retroviral therapy
CD4 +: CD4 positive T lymphocytes
CTL: Cytotoxic T-lymphocyte
CXCL10: (C-X-C Motif Chemokine Ligand 10
DNA: Deoxyribonucleic acid
HIV: Human immunodeficiency virus
HLA: Human leukocyte antigen
IFN-α2: Interferon-alpha2
IFN-γ: Interferon-gamma
IL-6: Interleukin-6
IL-8: Interleukin-8
IRIS: Immune Reconstitution Inflammatory Syndrome
KIR: Killer-cell immunoglobulin-like receptor
Mtb: *Mycobacterium tuberculosis*
NK Cells: Natural killer cells
OR: Odds ratio
PCRs: Polymerase chain reactions
TB: Tuberculosis
TNF: Tumor necrosis factor

## References

[1] WHO. Global Tuberculosis Report 2017: World Health Organization; 2017. p. 1–262. https://www.who.int/tb/publications/global_report/gtbr2017_main_text.pdf..

[2] MINISTÉRIO DA SAÚDE (2017). Plano Nacional pelo Fim da Tuberculose como Problema de Saúde Pública.

[3] Pean P, Nerrienet E, Madec Y, Borand L, Laureillard D, Fernandez M (2012). Natural killer cell degranulation capacity predicts early onset of the immune reconstitution inflammatory syndrome (IRIS) in HIV-infected patients with tuberculosis. Blood.

[4] Tan HY, Yong YK, Andrade BB, Shankar EM, Ponnampalavanar S, Omar SFS (2015). Plasma interleukin-18 levels are a biomarker of innate immune responses that predict and characterize tuberculosis-associated immune reconstitution inflammatory syndrome. AIDS.

[5] Vijaya Lakshmi V, Rakh SS, Anu Radha B, Hari Sai Priya V, Pantula V, Jasti S (2006). Role of HLA-B51 and HLA-B52 in susceptibility to pulmonary tuberculosis. Infect Genet Evol.

[6] Kulkarni S, Martin MP, Carrington M (2008). The Yin and Yang of HLA and KIR in human disease. Semin Immunol.

[7] Kaur G, Mehra N (2009). Genetic determinants of HIV-1 infection and progression to AIDS: immune response genes. Tissue Antigens.

[8] Kiepiela P, Leslie AJ, Honeyborne I, Ramduth D, Thobakgale C, Chetty S (2004). Dominant influence of HLA-B in mediating the potential co-evolution of HIV and HLA. Nature.

[9] Raghavan M, Geng J (2015). HLA-B polymorphisms and intracellular assembly modes. Mol Immunol.

[10] Colonna M, Borsellino G, Falco M, Ferrara GB, Strominger JL (1993). HLA-C is the inhibitory ligand that determines dominant resistance to lysis by NK1- and NK2-specific natural killer cells. Proc Natl Acad Sci U S A.

[11] Tallon BJM, Bruneau J, Tsoukas CM, Routy J-P, Kiani Z, Tan X (2014). Time to Seroconversion in HIV-Exposed Subjects Carrying Protective versus Non Protective KIR3DS1/L1 and HLA-B Genotypes. Sandberg JK, editor. PLoS One.

[12] Fernandes-Cardoso J, Süffert TA, M da G C, LFJ J, Jobim M, Salim PH (2016). Association between KIR genotypes and HLA-B alleles on viral load in Southern Brazilian individuals infected by HIV-1 subtypes B and C. Hum Immunol.

[13] Paximadis M, Minevich G, Winchester R, Schramm DB, Gray GE, Sherman GG (2011). KIR-HLA and Maternal-Infant HIV-1 Transmission in Sub-Saharan Africa. Sandberg J, editor. PLoS One.

[14] Gentle NL, Paximadis M, Puren A, Tiemessen CT (2013). Genetic Variability in Markers of HLA-C Expression in Two Diverse South African Populations. Ahuja SK, editor. PLoS One.

[15] Pydi SS, Sunder SR, Venkatasubramanian S, Kovvali S, Jonnalagada S, Valluri VL (2013). Killer cell immunoglobulin like receptor gene association with tuberculosis. Hum Immunol.

[16] Braun K, Wolfe J, Kiazyk S, Sharma MK (2015). Evaluation of host genetics on outcome of tuberculosis infection due to differences in killer immunoglobulin-like receptor gene frequencies and haplotypes. BMC Genet.

[17] Salie M, Daya M, Möller M, Hoal EG (2015). Activating KIRs alter susceptibility to pulmonary tuberculosis in a south African population. Tuberculosis.

[18] Habegger de Sorrentino A, Pardo R, Marinic K, Duarte SC, Lotero C (2014). KIR-HLA clase i y tuberculosis pulmonar en población amerindia del Chaco, Argentina. Enferm Infecc Microbiol Clin.

[19] Yuliwulandari R, Sachrowardi Q, Nakajima H, Kashiwase K, Hirayasu K, Mabuchi A (2010). Association of HLA-A, −B, and -DRB1 with pulmonary tuberculosis in western Javanese Indonesia. Hum Immunol.

[20] Figueiredo JF, Rodrigues Mde L, Deghaide NH, Donadi EA (2008). HLA profile in patients with AIDS and tuberculosis. Braz J Infect Dis.

[21] Müller M, Wandel S, Colebunders R, Attia S, Furrer H, Egger M (2010). Immune reconstitution inflammatory syndrome in patients starting antiretroviral therapy for HIV infection: a systematic review and meta-analysis. Lancet Infect Dis.

[22] Lai RPJ, Meintjes G, Wilkinson RJ (2016). HIV-1 tuberculosis-associated immune reconstitution inflammatory syndrome. Semin Immunopathol.

[23] Nelson AM, Manabe YC, Lucas SB (2017). Immune Reconstitution Inflammatory Syndrome (IRIS): What pathologists should know. Semin Diagn Pathol.

[24] Walker NF, Scriven J, Meintjes G, Wilkinson RJ (2015). Immune reconstitution inflammatory syndrome in HIV-infected patients. HIV AIDS (Auckl).

[25] Murdoch DM, Venter WD, Van Rie A, Feldman C (2007). Immune reconstitution inflammatory syndrome (IRIS): review of common infectious manifestations and treatment options. AIDS Res Ther.

[26] Gopal R, Rapaka RR, Kolls JK (2017). Immune reconstitution inflammatory syndrome associated with pulmonary pathogens. Eur Respir Rev.

[27] Lawn SD, Wood R (2005). Incidence of Tuberculosis during Highly Active Antiretroviral Therapy in High-Income and Low-Income Countries. Clin Infect Dis.

[28] Ganji R, Dhali S, Rizvi A, Rapole S, Banerjee S (2016). Understanding HIV-Mycobacteria synergism through comparative proteomics of intra-phagosomal mycobacteria during mono- and HIV co-infection. Sci Rep.

[29] Bana TM, Lesosky M, Pepper DJ, van der Plas H, Schutz C, Goliath R (2016). Prolonged tuberculosis-associated immune reconstitution inflammatory syndrome: characteristics and risk factors. BMC Infect Dis.

[30] Namale PE, Abdullahi LH, Fine S, Kamkuemah M, Wilkinson RJ, Meintjes G (2015). Paradoxical TB-IRIS in HIV-infected adults: a systematic review and meta-analysis. Future Microbiol.

[31] Serra FC, Hadad D, Orofino RL, Marinho F, Lourenço C, Morgado M (2007). Immune reconstitution syndrome in patients treated for HIV and tuberculosis in Rio de Janeiro. Braz J Infect Dis.

[32] da Silva TP, Giacoia-Gripp CBW, Schmaltz CA, Sant’ Anna FM, Rolla V, Morgado MG (2013). T Cell Activation and cytokine profile of Tuberculosis and HIV-positive Individuals during antituberculous treatment and Efavirenz-based regimens. PLoS One.

[33] Antonelli LRV, Mahnke Y, Hodge JN, Porter BO, Barber DL, Dersimonian R (2010). Elevated frequencies of highly activated CD4 T cells in HIV patients developing immune reconstitution inflammatory syndrome. Blood.

[34] Tran HTT, Van den Bergh R, Loembé MM, Worodria W, Mayanja-Kizza H, Colebunders R (2013). Modulation of the complement system in monocytes contributes to tuberculosis-associated immune reconstitution inflammatory syndrome. AIDS.

[35] Tran HTT, Van den Bergh R, Vu TN, Laukens K, Worodria W, Loembé MM (2014). The role of monocytes in the development of Tuberculosis-associated Immune Reconstitution Inflammatory Syndrome. Immunobiology.

[36] Affandi JS, Kumar M, Agarwal U, Singh S, Price P (2013). The search for a genetic factor associating with immune restoration disease in HIV patients co-infected with Mycobacterium tuberculosis. Dis Markers.

[37] Chang CC, Sheikh V, Sereti I, French MA (2014). Immune reconstitution disorders in patients with HIV infection: from pathogenesis to prevention and treatment. Curr HIV/AIDS Rep.

[38] Laureillard D, Marcy O, Madec Y, Chea S, Chan S, Borand L (2013). Paradoxical tuberculosis-associated immune reconstitution inflammatory syndrome after early initiation of antiretroviral therapy in a randomized clinical trial. AIDS.

[39] Luetkemeyer AF, Kendall MA, Nyirenda M, Wu X, Ive P, Benson CA (2014). Tuberculosis immune reconstitution inflammatory syndrome in A5221 STRIDE: timing, severity, and implications for HIV-TB programs. J Acquir Immune Defic Syndr.

[40] Meya DB, Manabe YC, Boulware DR, Janoff EN (2016). The immunopathogenesis of cryptococcal immune reconstitution inflammatory syndrome: understanding a conundrum. Curr Opin Infect Dis.

[41] Naidoo K, Yende-Zuma N, Padayatchi N, Naidoo K, Jithoo N, Nair G (2012). The immune reconstitution inflammatory syndrome after antiretroviral therapy initiation in patients with tuberculosis: findings from the SAPiT trial. Ann Intern Med.

[42] Goovaerts O, Jennes W, Massinga-Loembé M, Ondoa P, Ceulemans A, Vereecken C (2015). Lower Pre-Treatment T Cell Activation in Early- and Late-Onset Tuberculosis-Associated Immune Reconstitution Inflammatory Syndrome. Nixon DF, editor. PLoS One.

[43] Manosuthi W, Kiertiburanakul S, Phoorisri T, Sungkanuparph S (2006). Immune reconstitution inflammatory syndrome of tuberculosis among HIV-infected patients receiving antituberculous and antiretroviral therapy. J Inf Secur.

[44] Lai RPJ, Meintjes G, Wilkinson KA, Graham CM, Marais S, Van der Plas H (2015). HIV-tuberculosis-associated immune reconstitution inflammatory syndrome is characterized by Toll-like receptor and inflammasome signalling. Nat Commun.

[45] Oliver BG, Elliott JH, Price P, Phillips M, Saphonn V, Vun MC (2010). Mediators of innate and adaptive Immune responses differentially affect Immune restoration disease Associated with *Mycobacterium tuberculosis* in HIV Patients beginning antiretroviral therapy. J Infect Dis.

[46] da Silva TP, Giacoia-Gripp CBW, Schmaltz CA, Sant’Anna FM, Saad MH, de Matos JA (2017). Risk factors for increased immune reconstitution in response to Mycobacterium tuberculosis antigens in tuberculosis HIV-infected, antiretroviral-naïve patients. BMC Infect Dis.

[47] Giacoia-Gripp CBW, A da S C, da Silva TP, Sant’ Anna FM, CAS S, T de S B (2019). Changes in the NK Cell Repertoire Related to Initiation of TB Treatment and Onset of Immune Reconstitution Inflammatory Syndrome in TB/HIV Co-infected Patients in Rio de Janeiro, Brazil—ANRS 12274. Front Immunol.

[48] da Saúde M (2018). Protocolo Clínico e Diretrizes Terapêuticas para Manejo da Infecção pelo HIV em Adultos | Departamento de Doenças de Condições Crônicas e Infecções Sexualmente Transmissíveis [Internet].

[49] Saúde M DA (2011). Manual De Recomendações Para O Controle Da Tuberculose No Brasil.

[50] Robertson J, Meier M, Wall J, Ying J, Fichtenbaum CJ (2006). Immune reconstitution syndrome in HIV: validating a case definition and identifying clinical predictors in persons initiating antiretroviral therapy. Clin Infect Dis.

[51] Meintjes G, Lynen L (2008). Prevention and treatment of the immune reconstitution inflammatory syndrome. Curr Opin HIV AIDS.

[52] Demitto FO, Schmaltz CAS, Sant’Anna FM, Arriaga MB, Andrade BB, Rolla VC (2019). Predictors of early mortality and effectiveness of antiretroviral therapy in TB-HIV patients from Brazil. Nicastri E, editor. PLoS One.

[53] Robinson J, Halliwell JA, Mcwilliam H, Lopez R, SGE M (2013). IPD-the Immuno Polymorphism Database.

[54] Mandelboim O, Reyburn HT, Valés-Gómez M, Pazmany L, Colonna M, Borsellino G (1996). Protection from lysis by natural killer cells of group 1 and 2 specificity is mediated by residue 80 in human histocompatibility leukocyte antigen C alleles and also occurs with empty major histocompatibility complex molecules. J Exp Med.

[55] Faridi RM, Agrawal S (2011). Killer immunoglobulin-like receptors (KIRs) and HLA-C allorecognition patterns implicative of dominant activation of natural killer cells contribute to recurrent miscarriages. Hum Reprod.

[56] Uhrberg M, Valiante NM, Shum BP, Shilling HG, Lienert-Weidenbach K, Corliss B (1997). Human Diversity in Killer Cell Inhibitory Receptor Genes. Immunity.

[57] Agresti A (2002). An Introduction to Categorical Data Analysis.

[58] Fay MP, Feuer EJ (1997). Confidence Intervals For Directly Standardized Rates: A Method Based On The Gamma Distribution. Stat Med.

[59] Instituto Brasileiro de Geografia e Estatística (2013). Características étnico - raciais da população: classificação e identidades. Estudos e Análises: informação demográfica e socioeconômica.

[60] Gao X, O’Brien TR, Welzel TM, Marti D, Qi Y, Goedert JJ (2010). HLA-B alleles associate consistently with HIV heterosexual transmission, viral load, and progression to AIDS, but not susceptibility to infection. AIDS.

[61] Takejima P, Agondi RC, Rodrigues H, Aun MV, Kalil J, Giavina-Bianchi P (2017). Allergic and Nonallergic Asthma Have Distinct Phenotypic and Genotypic Features. Int Arch Allergy Immunol.

[62] Martin MP, Carrington M (2013). Immunogenetics of HIV disease. Immunol Rev.

[63] Stein CM, Sausville L, Wejse C, Sobota RS, Zetola NM, Hill PC (2017). Genomics of human pulmonary tuberculosis: from genes to pathways. Curr Genet Med Rep.

[64] Wilkinson KA, Walker N (2016). Biomarkers for identifying risk of Immune Reconstitution Inflammatory Syndrome. EBioMed.

[65] George V, Harrison L, Roach M, Li X-D, Tierney C, Fischl MA (2017). Associations of plasma cytokine and microbial translocation biomarkers with Immune Reconstitution Inflammatory Syndrome. J Infect Dis.

[66] Conesa-Botella A, Meintjes G, Coussens AK, Van Der Plas H, Goliath R, Schutz C (2012). Corticosteroid therapy, vitamin D status, and inflammatory cytokine profile in the HIV-tuberculosis immune reconstitution inflammatory syndrome. Clin Infect Dis.

[67] Bourgarit A, Carcelain G, Samri A, Parizot C, Lafaurie M, Abgrall S (2009). Tuberculosis-associated immune restoration syndrome in HIV-1-infected patients involves tuberculin-specific CD4 Th1 cells and KIR-negative gammadelta T cells. J Immunol.

[68] Conradie (2011). Natural Killer cell activation distinguishes M. tuberculosis-mediated Immune reconstitution syndrome (IRIS) from chronic HIV and HIV-MTB co-infection. J Acquir Immune Defic Syndr.

[69] Teixeira SLM, de Sá NBR, Campos DP, Coelho AB, Guimarães ML, Leite TCNF (2014). Association of the HLA-B*52 allele with non-progression to AIDS in Brazilian HIV-1-infected individuals. Genes Immun.

[70] Halagan M, Oliveira DC, Maiers M, Fabreti-Oliveira RA, Moraes MEH, Visentainer JEL (2018). The distribution of HLA haplotypes in the ethnic groups that make up the Brazilian Bone Marrow Volunteer Donor Registry (REDOME). Immunogenetics.

[71] Ayo CM, da Silveira CAV, Xavier DH, Batista MF, Carneiro OA, Brandão de Mattos CC (2015). Frequencies of allele groups HLA-A, HLA-B and HLA-DRB1 in a population from the northwestern region of São Paulo state, Brazil. Int J Immunogenet.

[72] Torres L, da Silva Bouzas LF, Almada A, de Sobrino Porto LCM, Abdelhay E (2017). Distribution of HLA-A, −B and -DRB1 antigenic groups and haplotypes from the Brazilian bone marrow donor registry (REDOME). Hum Immunol.

[73] Rudnick CCC, Franceschi DSA, Marangon AV, Guelsin GAS, Sell AM, Visentainer JEL (2008). Killer cell immunoglobulin-like receptor gene diversity in a southern Brazilian population from the state of Paraná. Hum Immunol.

[74] Jobim M, Salim PH, Portela P, Wilson TJ, Fraportti J, Baronio D (2010). Killer cell immunoglobulin-like receptor gene diversity in a Caucasian population of southern Brazil. Int J Immunogenet.

[75] Augusto DG, Zehnder-Alves L, Pincerati MR, Martin MP, Carrington M, Petzl-Erler ML (2012). Diversity of the KIR gene cluster in an urban Brazilian population. Immunogenetics.

[76] McNeil AJ, Yap PL, Gore SM, Brettle RP, McColl M, Wyld R (1996). Association of HLA types A1-B8-DR3 and B27 with rapid and slow progression of HIV disease. QJM.

[77] Carrington M, O’Brien SJ (2003). The Influence of *HLA* Genotype on AIDS. Annu Rev Med.

[78] Tan HY, Yong YK, Shankar EM, Paukovics G, Ellegård R, Larsson M (2016). Aberrant Inflammasome Activation Characterizes Tuberculosis-Associated Immune Reconstitution Inflammatory Syndrome. J Immunol.

[79] Shankarkumar U, Pawar A, Prabu G, Ghosh K (2009). Role of HLA class i (HLA-A, B) and HLA class II (HLA-DRB, DQB) in HIV-1 patients with and without pulmonary tuberculosis. J Acquir Immune Defic Syndr.

[80] McClelland EE, Smith JM (2011). Gender Specific Differences in the Immune Response to Infection. Arch Immunol Ther Exp.

[81] Klein SL (2004). Hormonal and immunological mechanisms mediating sex differences in parasite infection. Parasite Immunol.

[82] Herzmann C, Sotgiu G, Bellinger O, Diel R, Gerdes S, Goetsch U (2017). Risk for latent and active tuberculosis in Germany. Infection.

[83] Pegram HJ, Andrews DM, Smyth MJ, Darcy PK, Kershaw MH (2011). Activating and inhibitory receptors of natural killer cells. Immunol Cell Biol.

[84] Alter G, Heckerman D, Schneidewind A, Fadda L, Kadie CM, Carlson JM (2011). HIV-1 adaptation to NK-cell-mediated immune pressure. Nature.

[85] Gooneratne SL, Richard J, Lee WS, Finzi A, Kent SJ, Parsons MS (2015). Slaying the Trojan horse: natural killer cells exhibit robust anti-HIV-1 antibody-dependent activation and cytolysis against allogeneic T cells. J Virol.

[86] Misra MK, Prakash S, Moulik NR, Kumar A, Agrawal S (2016). Genetic associations of killer immunoglobulin like receptors and class I human leukocyte antigens on childhood acute lymphoblastic leukemia among north Indians. Hum Immunol.

[87] Barcelos W, Sathler-Avelar R, Martins-Filho OA, Carvalho BN, Guimarães TMPD, Miranda SS (2008). Natural Killer Cell Subpopulations in Putative Resistant Individuals and Patients with Active Mycobacterium tuberculosis Infection. Scand J Immunol.

[88] Bozzano F, Costa P, Passalacqua G, Dodi F, Ravera S, Pagano G (2009). Functionally relevant decreases in activatory receptor expression on NK cells are associated with pulmonary tuberculosis in vivo and persist after successful treatment. Int Immunol.

[89] Portevin D, Via LE, Eum S, Young D (2012). Natural killer cells are recruited during pulmonary tuberculosis and their *ex vivo* responses to mycobacteria vary between healthy human donors in association with KIR haplotype. Cell Microbiol.

[90] Méndez A, Granda H, Meenagh A, Contreras S, Zavaleta R, Mendoza MF (2006). Study of KIR genes in tuberculosis patients. Tissue Antigens.

[91] Mahfouz R, Halas H, Hoteit R, Saadeh M, Shamseddeen W, Charafeddine K (2011). Study of KIR genes in Lebanese patients with tuberculosis. Int J Tuberc Lung Dis.

[92] Biberg-Salum TG, M de L VR, APM F, NHS D, de PJS, Castelli EC (2018). *HLA-C* Alleles and Cytomegalovirus Retinitis in Brazilian Patients with AIDS. J Ophthalmol.

[93] Shelburne SA, Visnegarwala F, Darcourt J, Graviss EA, Giordano TP, White AC, et al. Incidence and risk factors for immune reconstitution inflammatory syndrome during highly active antiretroviral therapy. 2005;19(4):399 Available from: https://insights.ovid.com/pubmed?pmid=15750393. [Cited 2018 Aug 22]. 10.1097/01.aids.0000161769.06158.8a 15750393

[94] Srivastava RM, Khar A. Activating and inhibitory receptors and their role in Natural Killer cell function. Indian J Biochem Biophys. 2003;40 Available from: https://pdfs.semanticscholar.org/a6e5/ac54c8523630062bab92a4ac507e20f9c05e.pdf. [Cited 2018 Sep 3]. 22900322

[95] Long EO. Negative signalling by inhibitory receptors: the NK cell paradigm. Available from: https://www.ncbi.nlm.nih.gov/pmc/articles/PMC2587243/pdf/nihms47673.pdf. [Cited 2018 Sep 3] 10.1111/j.1600-065X.2008.00660.x PMC2587243 18759921

[96] Salim PH, Jobim M, Jobim LF, Xavier RM (2011). Doenças reumatológicas autoimunes e sua associação com os genes killer immunoglobulin-like receptors. Rev Bras Reumatol.

