# Supplementary material for: Clinical and genetic markers associated with tuberculosis, HIV-1 infection, and TB/HIV-immune reconstitution inflammatory syndrome outcomes
Source: BMC Infect Dis. 2020 Jan 20;20:59. doi: 10.1186/s12879-020-4786-5 (PMC6971853; doi:10.1186/s12879-020-4786-5)
Supplement: Supplementary file 1 — Additional file 1: Table S1. Sociodemographic, clinical, and laboratory data of HIV-1-positive individuals included in the study categorized according to the presence (G1) or absence (G2) of TB. Table S2. Distribution of HLA-B, HLA-C, and KIR genetic profiles found in this study. Table S3. Distribution of HLA-B and HLA-C serological epitopes of subjects included in this study stratified by groups. Table S4. Distribution of HLA-B alleles of the subjects included in this study and the Brazilian general population (data from the Brazilian Registry of Bone Marrow Donors - REDOME). Table S5. Frequency of KIR genotypes and mapping of the inhibitory/activator genes among the subjects included in this study. Table S6. Distribution of KIR genotypes of subjects included in this study stratified by groups. Table S7. Sociodemographic, clinical, and laboratory data of HIV-TB individuals with and without IRIS. [file 12879_2020_4786_MOESM1_ESM.docx]

**Additional file 1**

Table S1: Sociodemographic, clinical, and laboratory data of HIV-1-positive individuals included in the study categorized according to the presence (G1) or absence (G2) of TB.

| **Features** | **Overall**  **N=112** | | **HIV-1 positive individuals** | | ***P*-value^a^** |
| --- | --- | --- | --- | --- | --- |
|  |  |  | **With TB (G1)**  **N=88** | **Without TB (G2)**  **N=24** |  |
| **Gender; n (%)** | |  |  |  |  |
| **Female** | 26 (23.2) | | 18 (20.5) | 8 (33.3) | 0.274 |
| **Male** | 86 (76.8) | | 70 (79.54) | 16 (66.7) |  |
| **SkinColor; n (%)** | |  |  |  |  |
| **Black** | 25 (22.3) | | 21 (23.9) | 4 (16.7) | 0.202 |
| **Brown** | 46 (41.1) | | 32 (36.4) | 14 (58.3) |  |
| **White** | 41 (36.6) | | 35 (39.7) | 6 (25) |  |
| **Education^b^; n (%)** | |  |  |  |  |
| **Bachelor** | 4 (3.6) | | 3 (3.4) | 1 (4.2) | **0.011** |
| **Upper-secondary** | 28 (25) | | 19 (21.6) | 9 (37.5) |  |
| **Lower-secondary** | 52 (46.4) | | 38 (43.2) | 14 (58.3) |  |
| **Primary** | 23 (20.5) | | 23 (26.1) | 0 (0) |  |
| **Unknown** | 5 (4.5) | | 5 (5.7) | 0 (0) |  |
| **HIV transmission route; n (%)** | |  |  |  |  |
| **Heterosexual** | 84 (75) | | 66 (75) | 18 (75) | 1 |
| **MSM** | 26 (23.2) | | 20 (22.7) | 6 (25) |  |
| **Unknown** | 2 (1.8) | | 2 (2.3) | 0 (0) |  |
| **Clinical Parameters** | |  |  |  |  |
| **CD4 count (cell/µL) (IQR)** | 89 (122) | | 89 (101.1) | 67.5 (281.2) | 0.170 |
| **(≤50]; n(%)** | 40 (37) | | 28 (31.8) | 12 (50) | 0.155 |
| **(>50]; n(%)** | 68 (63) | | 56 (63.6) | 12 (50) |  |
| **CD8 (IQR)** | 591 (609) | | 599 (680.7) | 462 (1925) | 0.205 |
| **(≤500]; n(%)** | 43 (41.7) | | 29 (32.9) | 14 (58.3) | 0.097 |
| **(>500]; n(%)** | 60 (58.3) | | 50 (56.8) | 10 (8.9) |  |
| **CD4/CD8 (IQR)** | 0.13 (0.24) | | 0.13 (0.15) | 0.13 (0.13) | 0.466 |
| **(≤0.15]; n(%)** | 60 (58.3) | | 46 (52.3) | 14 (12.5) | 1 |
| **(>0.15]; n(%)** | 43 (41.7) | | 33 (37.5) | 10 (41.7) |  |
| **Viral load (copies/mL) (IQR)** | 195,969 (421,635) | | 221,184 (444,669) | 166,369 (247,473) | 0.985 |
| **(≤20000]; n(%)** | 52 (50) | | 39 (44.3) | 13 (54.2) | 0.816 |
| **(>20000]; n(%)** | 52 (50) | | 41 (46.6) | 11 (45.8) |  |
| **Log VL (IQR)** | 5.29 (0.86) | | 5.34 (6.06) | 5.22 (21.75) | 0.985 |

^a^P-values were calculated using Fisher’s exact test. Differences were considered significant with a value of * P < 0.05. ^b^Classification, according to the International Standard Classification of Education (ISCED) maintained by the United Nations Educational, Scientific and Cultural Organization (UNESCO). N: number of individuals in each group; TB: tuberculosis; IQR: interquartile range; VL: viral load.

Table S2: Distribution of HLA-B, HLA-C, and KIR genetic profiles found in this study.

| **HLA and KIR genes** | **All the Groups** | | **G1** | | **G2** | | | | **G3** | | | | | **G4** | | | | | **With IRIS** | | | | | **Without IRIS** | | | | |
| --- | --- | --- | --- | --- | --- | --- | --- | --- | --- | --- | --- | --- | --- | --- | --- | --- | --- | --- | --- | --- | --- | --- | --- | --- | --- | --- | --- | --- |
|  | **N=162** | | **N=88** | | **N=24** | | | | **N=24** | | | | | **N=26** | | | | | **N=11** | | | | | **N=77** | | | | |
| **HLA-B alleles** | **n** | **Freq** | **n** | **Freq** | **n** | | | **Freq** | **n** | | | | **Freq** | **n** | | | | **Freq** | **n** | | | | **Freq** | **n** | | | **Freq** | |
| **B*07** | 23 | 14.2% | 15 | 17.0% | 4 | | | 16.7% | 2 | | | | 8.3% | 2 | | | | 7.7% | 3 | | | | 27.3% | 12 | | | 15.6% | |
| **B*08** | 16 | 9.9% | 6 | 6.8% | 3 | | | 12.5% | 1 | | | | 4.2% | 6 | | | | 23.1% | 0 | | | | 0% | 6 | | | 7.8% | |
| **B*13** | 5 | 3.1% | 1 | 1.1% | 3 | | | 12.5% | 0 | | | | 0% | 1 | | | | 3.8% | 0 | | | | 0% | 1 | | | 1.3% | |
| **B*14** | 18 | 11.1% | 8 | 9.1% | 5 | | | 20.8% | 4 | | | | 16.7% | 1 | | | | 3.8% | 1 | | | | 9.1% | 7 | | | 9.1% | |
| **B*15** | 43 | 26.5% | 31 | 35.2% | 6 | | | 25.0% | 4 | | | | 16.7% | 2 | | | | 7.7% | 3 | | | | 27.3% | 28 | | | 36.4% | |
| **B*18** | 9 | 5.6% | 3 | 3.4% | 1 | | | 4.2% | 2 | | | | 8.3% | 3 | | | | 11.5% | 1 | | | | 9.1% | 2 | | | 2.6% | |
| **B*21** | 1 | 0.6% | 1 | 1.1% | 0 | | | 0% | 0 | | | | 0% | 0 | | | | 0% | 0 | | | | 0% | 1 | | | 1.3% | |
| **B*27** | 3 | 1.9% | 3 | 3.4% | 0 | | | 0% | 0 | | | | 0% | 0 | | | | 0% | 0 | | | | 0% | 3 | | | 3.9% | |
| **B*35** | 30 | 18.5% | 19 | 21.6% | 2 | | | 8.3% | 4 | | | | 16.7% | 5 | | | | 19.2% | 2 | | | | 18.2% | 17 | | | 22.1% | |
| **B*37** | 2 | 1.2% | 2 | 2.3% | 0 | | | 0% | 0 | | | | 0% | 0 | | | | 0% | 0 | | | | 0% | 2 | | | 2.6% | |
| **B*38** | 8 | 4.9% | 3 | 3.4% | 0 | | | 0% | 0 | | | | 0% | 5 | | | | 19.2% | 1 | | | | 9.1% | 2 | | | 2.6% | |
| **B*39** | 8 | 4.9% | 3 | 3.4% | 2 | | | 8.3% | 3 | | | | 12.5% | 0 | | | | 0% | 1 | | | | 9.1% | 2 | | | 2.6% | |
| **B*40** | 8 | 4.9% | 5 | 5.7% | 1 | | | 4.2% | 1 | | | | 4.2% | 1 | | | | 3.8% | 1 | | | | 9.1% | 4 | | | 5.2% | |
| **B*41** | 5 | 3.1% | 3 | 3.4% | 0 | | | 0% | 1 | | | | 4.2% | 1 | | | | 3.8% | 1 | | | | 9.1% | 2 | | | 2.6% | |
| **B*42** | 9 | 5.6% | 4 | 4.5% | 0 | | | 0% | 3 | | | | 12.5% | 2 | | | | 7.7% | 2 | | | | 18.2% | 2 | | | 2.6% | |
| **B*44** | 33 | 20.4% | 18 | 20.5% | 4 | | | 16.7% | 5 | | | | 20.8% | 6 | | | | 23.1% | 2 | | | | 18.2% | 16 | | | 20.8% | |
| **B*45** | 9 | 5.6% | 4 | 4.5% | 2 | | | 8.3% | 3 | | | | 12.5% | 0 | | | | 0% | 1 | | | | 9.1% | 3 | | | 3.9% | |
| **B*48** | 4 | 2.5% | 2 | 2.3% | 0 | | | 0% | 1 | | | | 4.2% | 1 | | | | 3.8% | 0 | | | | 0% | 2 | | | 2.6% | |
| **B*49** | 6 | 3.7% | 4 | 4.5% | 2 | | | 8.3% | 0 | | | | 0% | 0 | | | | 0% | 1 | | | | 9.1% | 3 | | | 3.9% | |
| **B*50** | 4 | 2.5% | 2 | 2.3% | 0 | | | 0% | 1 | | | | 4.2% | 1 | | | | 3.8% | 0 | | | | 0% | 2 | | | 2.6% | |
| **B*51** | 16 | 9.9% | 7 | 8.0% | 3 | | | 12.5% | 2 | | | | 8.3% | 4 | | | | 15.4% | 0 | | | | 0% | 7 | | | 9.1% | |
| **B*52** | 8 | 4.9% | 4 | 4.5% | 1 | | | 4.2% | 1 | | | | 4.2% | 2 | | | | 7.7% | 0 | | | | 0% | 4 | | | 5.2% | |
| **B*53** | 19 | 11.7% | 10 | 11.4% | 5 | | | 20.8% | 3 | | | | 12.5% | 1 | | | | 3.8% | 1 | | | | 9.1% | 9 | | | 11.7% | |
| **B*55** | 1 | 0.6% | 1 | 1.1% | 0 | | | 0% | 0 | | | | 0% | 0 | | | | 0% | 0 | | | | 0% | 1 | | | 1.3% | |
| **B*56** | 1 | 0.6% | 0 | 0% | 0 | | | 0% | 0 | | | | 0% | 1 | | | | 3.8% | 0 | | | | 0% | 0 | | | 0.% | |
| **B*57** | 11 | 6.8% | 5 | 5.7% | 2 | | | 8.3% | 3 | | | | 12.5% | 1 | | | | 3.8% | 1 | | | | 9.1% | 4 | | | 5.2% | |
| **B*58** | 16 | 9.9% | 8 | 9.1% | 2 | | | 8.3% | 2 | | | | 8.3% | 4 | | | | 15.4% | 0 | | | | 0% | 8 | | | 10.4% | |
| **B*78** | 1 | 0.6% | 1 | 1.1% | 0 | | | 0% | 0 | | | | 0% | 0 | | | | 0% | 0 | | | | 0% | 1 | | | 1.3% | |
| **B*81** | 6 | 3.7% | 3 | 3.4% | 0 | | | 0% | 1 | | | | 4.2% | 2 | | | | 7.7% | 0 | | | | 0% | 3 | | | 3.9% | |
| **B*82** | 1 | 0.6% | 0 | 0% | 0 | | | 0% | 1 | | | | 4.2% | 0 | | | | 0% | 0 | | | | 0% | 0 | | | 0% | |
| **HLA-C1 alleles^a^** |  |  |  |  |  | | |  |  | | | |  |  | | | |  |  | | | |  |  | | |  | |
| **C*01** | 10 | 6.3% | 7 | 8.1% | 0 | | | 0.% | 1 | | | | 4.2% | 2 | | | | 7.7% | 0 | | | | 0.0% | 7 | | | 9.3% | |
| **C*03** | 30 | 18.8% | 18 | 20.9% | 4 | | | 16.7% | 6 | | | | 25.0% | 2 | | | | 7.7% | 3 | | | | 27.3% | 15 | | | 20.0% | |
| **C*07** | 64 | 40.0% | 30 | 34.9% | 14 | | | 58.3% | 11 | | | | 45.8% | 9 | | | | 34.6% | 5 | | | | 45.5% | 25 | | | 33.3% | |
| **C*08** | 16 | 10.0% | 8 | 9.3% | 3 | | | 12.5% | 3 | | | | 12.5% | 2 | | | | 7.7% | 1 | | | | 9.1% | 7 | | | 9.3% | |
| **C*12** | 15 | 9.4% | 8 | 9.3% | 1 | | | 4.2% | 1 | | | | 4.2% | 5 | | | | 19.2% | 2 | | | | 18.2% | 6 | | | 8.0% | |
| **C*14** | 6 | 3.8% | 2 | 2.3% | 2 | | | 8.3% | 0 | | | | 0% | 2 | | | | 7.7% | 0 | | | | 0% | 2 | | | 2.7% | |
| **C*16** | 21 | 13.1% | 10 | 11.6% | 5 | | | 20.8% | 3 | | | | 12.5% | 3 | | | | 11.5% | 3 | | | | 27.3% | 7 | | | 9.3% | |
| **HLA-C2 alleles^a^** |  |  |  |  |  | | |  |  | | | |  |  | | | |  |  | | | |  |  | | |  | |
| **C*02** | 22 | 13.8% | 15 | 17.4% | 4 | | | 16.7% | 0 | | | | 0% | 3 | | | | 11.5% | 2 | | | | 18.2% | 13 | | | 17.3% | |
| **C*04** | 60 | 37.5% | 36 | 41.9% | 8 | | | 33.3% | 6 | | | | 25.0% | 10 | | | | 38.5% | 3 | | | | 27.3% | 33 | | | 44.0% | |
| **C*05** | 20 | 12.5% | 8 | 9.3% | 1 | | | 4.2% | 6 | | | | 25.0% | 5 | | | | 19.2% | 0 | | | | 0% | 8 | | | 10.7% | |
| **C*06** | 31 | 19.4% | 17 | 19.8% | 4 | | | 16.7% | 6 | | | | 25.0% | 4 | | | | 15.4% | 1 | | | | 9.1% | 16 | | | 21.3% | |
| **C*15** | 7 | 4.4% | 4 | 4.7% | 0 | | | 0% | 2 | | | | 8.3% | 1 | | | | 3.8% | 0 | | | | 0% | 4 | | | 5.3% | |
| **C*17** | 10 | 6.3% | 5 | 5.8% | 0 | | | 0% | 2 | | | | 8.3% | 3 | | | | 11.5% | 2 | | | | 18.2% | 3 | | | 4.0% | |
| **C*18** | 8 | 5.0% | 4 | 4.7% | 2 | | | 8.3% | 1 | | | | 4.2% | 1 | | | | 3.8% | 0 | | | | 0% | 4 | | | 5.3% | |
| **Activating KIR genes** | | | | | | | | | | | | | | | | | | | | | | | | | | | | |
| **2DL1** | 157 | 96.9% | 85 | 96.6% | 23 | | | 95.8% | 24 | | | | 100% | 25 | | | | 96.2% | 11 | | | | 100% | 74 | | | | 96.1% |
| **2DL2** | 90 | 55.6% | 50 | 56.8% | 11 | | | 45.8% | 16 | | | | 66.7% | 13 | | | | 50% | 9 | | | | 81.8% | 41 | | | | 53.2% |
| **2DL3** | 149 | 91.9% | 84 | 95.5% | 19 | | | 79.2% | 21 | | | | 87.5% | 25 | | | | 96.2% | 11 | | | | 100% | 73 | | | | 94.8% |
| **2DL4** | 162 | 100% | 88 | 100% | 24 | | | 100% | 24 | | | | 100% | 26 | | | | 100% | 11 | | | | 100% | 77 | | | | 100% |
| **2DL5** | 75 | 46.3% | 36 | 40.9% | 13 | | | 54.2% | 16 | | | | 66.7% | 10 | | | | 38.5% | 5 | | | | 45.5% | 31 | | | | 40.3% |
| **3DL1** | 158 | 97.5% | 86 | 97.7% | 22 | | | 91.7% | 24 | | | | 100% | 26 | | | | 100% | 11 | | | | 100% | 75 | | | | 97.4% |
| **3DL2** | 161 | 99.4% | 87 | 98.9% | 24 | | | 100% | 24 | | | | 100% | 25 | | | | 100% | 11 | | | | 100% | 76 | | | | 98.7% |
| **3DL3** | 161 | 99.4% | 88 | 100% | 24 | | | 100% | 24 | | | | 100% | 8 | | | | 96.2% | 11 | | | | 100% | 77 | | | | 100% |
| **Inhibitory KIR genes** | | | | | | | | | | | | | | | | | | | | | | | | | | | | |
| **2DS1** | 61 | 37.6% | 31 | 36.4% | 12 | | | 50% | 10 | | | | 41.7% | 7 | | | | 26.9% | 7 | | | | 63.6% | 25 | | | | 32.5% |
| **2DS2** | 83 | 51.2% | 46 | 52.3% | 14 | | | 41.7% | 16 | | | | 66.7% | 11 | | | | 42.3% | 9 | | | | 81.8% | 37 | | | | 48.1% |
| **2DS3** | 43 | 26.5% | 20 | 22.7% | 8 | | | 33.3% | 10 | | | | 41.7% | 5 | | | | 19.2% | 3 | | | | 27.3% | 17 | | | | 22.1% |
| **2DS4** | 157 | 96.9% | 85 | 96.6% | 22 | | | 91.7% | 24 | | | | 100% | 26 | | | | 100% | 11 | | | | 100% | 74 | | | | 96.1% |
| **2DS5** | 66 | 40.7% | 34 | 38.6% | 13 | | | 54.2% | 11 | | | | 45.8% | 8 | | | | 30.8% | 7 | | | | 63.6% | 27 | | | | 35.1% |
| **3DS1** | 54 | 33.3% | 28 | 31.8% | 9 | | | 37.5% | 9 | | | | 37.5% | 25 | | | | 30.8% | 5 | | | | 45.5% | 23 | | | | 29.9% |
| **Pseudogenes KIR** |  |  |  |  |  | | |  |  | | | |  |  | | | |  |  | | | |  |  | | | |  |
| **2DP1** | 157 | 96.9% | 85 | 96.6% | 23 | | | 95.8% | 24 | | | | 100% | 26 | | | | 96.2% | 11 | | | | 100% | 74 | | | | 96.1% |
| **3DP1** | 161 | 99.4% | 87 | 98.9% | 24 | | | 100% | 24 | | | | 100% | 26 | | | | 100% | 11 | | | | 100% | 76 | | | | 98.7% |
| **HLA-B serological epitopes** | | | | | | | | | | | | | | | | | | | | | | | | | | | | |
| **Epitope Bw4/Bw4** | 24 | 14.8% | 12 | 13.6% | 4 | | | 16.6% | 2 | | | | 8.3% | 6 | | | 23.1% | | 0 | | | 0% | | 12 | | | 11.7% | |
| **Epitope Bw6/Bw6** | 53 | 32.7% | 31 | 35.2% | 6 | | | 25% | 9 | | | 37.5% | | 7 | | | 26.9% | | 5 | | | 36.4% | | 26 | | | 37.7% | |
| **Epitope Bw4/Bw6** | 85 | 52.5% | 45 | 51.1% | 14 | | | 58.3% | 13 | | | 54.1% | | 13 | | | 50% | | 6 | | | 63.6% | | 39 | | | 50.6% | |
| **HLA-C serological epitopes^a^** | | | | | | | | | | | | | | | | | | | | | | | | | | | | |
| **Epitope C1/C1** | 43 | 26.9% | 21 | 24.4% | 10 | | | 41.7% | 7 | | | 41.7% | | 5 | | | 19.2% | | 6 | | | 54.5% | | 15 | | | 20.0% | |
| **Epitope C2/C2** | 41 | 25.6% | 24 | 27.9% | 5 | | | 20.8% | 6 | | | 20.8% | | 6 | | | 23.1% | | 3 | | | 27.3% | | 21 | | | 28.0% | |
| **Epitope C1/C2** | 76 | 47.5% | 41 | 47.7% | 9 | | | 37.5% | 11 | | | 37.5% | | 15 | | | 57.7% | | 2 | | | 18.2% | | 39 | | | 52.0% | |
| **KIR Genotypes** | | | | | | | | | | | | | | | | | | | | | | | | | | | | |
| **Genotypes AA** | 50 | 30.9% | 26 | 29.5% | 8 | | 33.3% | | 6 | | 25.0% | | | 10 | | 38.5% | | | 1 | | 9.1% | | | 25 | | 32.5% | | |
| **Genotypes Bx** | 112 | 69.1% | 62 | 70.4% | 16 | | 66.7% | | 18 | | 75.0% | | | 16 | | 61.5% | | | 10 | | 90.9% | | | 52 | | 67.5% | | |
| **KIR combined genotypes+HLA-B** | | | | | | | | | | | | | | | | | | | | | | | | | | | | |
| **KIR3DL1+Bw4/Bw4** | 23 | 14.2% | 2 | 13.6% | 3 | 12.5% | | | 2 | 8.3% | | | | 6 | 23.1% | | | | 0 | 0% | | | | 12 | 15.6% | | | |
| **KIR3DL1+Bw4/Bw6** | 84 | 51.9% | 45 | 51.1% | 13 | 54.2% | | | 13 | 54.2% | | | | 13 | 50% | | | | 6 | 54.5% | | | | 39 | 50.6% | | | |
| **KIR3DL1+Bw6/Bw6** | 51 | 31.5% | 29 | 33% | 6 | 25% | | | 9 | 37.5% | | | | 7 | 26.9% | | | | 5 | 45.5% | | | | 24 | 31.2% | | | |
| **KIR3DS1+Bw4/Bw4** | 5 | 3.1% | 2 | 2.3% | 1 | 4.2% | | | 0 | 0% | | | | 2 | 7.7% | | | | 0 | 0% | | | | 2 | 2.6% | | | |
| **KIR3DS1+Bw4/Bw6** | 30 | 18.5% | 15 | 17% | 7 | 29.2% | | | 6 | 25.0% | | | | 2 | 7.7% | | | | 3 | 27.3% | | | | 12 | 15.6% | | | |
| **KIR3DS1+Bw6/Bw6** | 19 | 11.7% | 11 | 12.5% | 1 | 4.2% | | | 3 | 12.5% | | | | 4 | 15.4% | | | | 2 | 18.2% | | | | 9 | 11.7% | | | |
| **KIR combined genotypes+HLA-C** | | | | | | | | | | | | | | | | | | | | | | | | | | | | |
| **KIR2DL1+C2/C2** | 39 | 24.4% | 23 | 26.7% | 5 | 20.8% | | | 6 | 25.0% | | | | 5 | 19.2% | | | | 3 | 27.3% | | | | 20 | 26.7% | | | |
| **KIR2DL1+C1/C2** | 75 | 46.9% | 40 | 46.5% | 9 | 37.5% | | | 11 | 45.8% | | | | 15 | 57.7% | | | | 2 | 18.2% | | | | 38 | 50.7% | | | |
| **KIR2DL2+C1/C1** | 26 | 16.3% | 15 | 17.4% | 6 | 25.0% | | | 4 | 16.7% | | | | 1 | 3.8% | | | | 4 | 36.4% | | | | 11 | 14.7% | | | |
| **KIR2DL2+C1/C2** | 38 | 23.8% | 18 | 20.9% | 2 | 8.3% | | | 9 | 37.5% | | | | 9 | 34.6% | | | | 1 | 9.1% | | | | 16 | 21.3% | | | |
| **KIR2DL3+C1/C1** | 38 | 23.8% | 20 | 23.3% | 7 | 29.2% | | | 6 | 25.0% | | | | 5 | 19.2% | | | | 6 | 54.5% | | | | 14 | 18.7% | | | |
| **KIR2DL3+C1/C2** | 73 | 45.6% | 40 | 46.5% | 9 | 37.5% | | | 9 | 37.5% | | | | 15 | 57.7% | | | | 2 | 18.2% | | | | 38 | 50.7% | | | |
| **KIR2DS1+C2/C2** | 17 | 10.6% | 11 | 12.8% | 3 | 12.5% | | | 2 | 8.3% | | | | 1 | 3.8% | | | | 3 | 27.3% | | | | 8 | 10.7% | | | |
| **KIR2DS1+C1/C2** | 25 | 15.6% | 12 | 14.0% | 3 | 12.5% | | | 5 | 20.8% | | | | 5 | 19.2% | | | | 1 | 9.1% | | | | 11 | 14.7% | | | |
| **KIR2DS2+C1/C1** | 25 | 15.6% | 14 | 16.3% | 6 | 25.0% | | | 4 | 16.7% | | | | 1 | 3.8% | | | | 4 | 36.4% | | | | 10 | 13.3% | | | |
| **KIR2DS2+C1/C2** | 34 | 21.3% | 16 | 18.6% | 1 | 4.2% | | | 9 | 37.5% | | | | 8 | 30.8% | | | | 2 | 18.2% | | | | 14 | 18.7% | | | |

^a^The HLA-C determination was not possible for two individuals from G1. So, when considering this variable, N (G1) = 86 and N (Without IRIS) = 75. NA: not applicable; N: number of individuals in each group; n: observed number of an allele/genetic marker carriers; Freq: relative frequency of carriers for each allele/genetic marker, calculated as the number of carriers/N.

Table S3: Distribution of HLA-B and HLA-C serological epitopes of subjects included in this study stratified by groups.

| HLAserological epitopes | All the groups | | | | HIV-1 positive individuals | | | | IRIS | | | |
| --- | --- | --- | --- | --- | --- | --- | --- | --- | --- | --- | --- | --- |
|  | **With TB (G1+G3)** | **Without TB (G2+G4)** | **aOR^a^**  **(95% CI)** | **p-value^b^** | **With TB (G1)** | **Without TB (G2)** | **aOR**  **(95% CI)** | **p-valor** | **Without IRIS** | **With IRIS** | **aOR**  **(95% CI)** | **p-value** |
|  | **N=50** | **N=112** |  |  | **N=88** | **N=24** |  |  | **N=77** | **N=11** |  |  |
| **HLA-B** | | | | | | | | | | | | |
| **Epitope Bw6/Bw4** | 58 (51.79) | 27 (54) | Ref | | 45 (51.14) | 14 (58.33) | Ref | | 39 (50.65) | 6 (54.55) | Ref | |
| **Epitope Bw4/Bw4** | 14 (12.5) | 10 (20) | 0.58  (0.17-1.97) | 0.383 | 12 (13.64) | 4 (16.67) | 1.31 (0.31-5.5) | 0.715 | 12 (15.58) | 0 (0) | NC | |
| **Epitope Bw6/Bw6** | 40 (35.71) | 13 (26) | 1.87 (0.73-4.76) | 0.192 | 31 (35.23) | 6 (25) | 1.62 (0.52-5.04) | 0.408 | 26 (33.77) | 5 (45.45) | 0.88 (0.15-5.35) | 0.894 |
| **HLA-C^c^** | | | | | | | | | | | | |
| **Epitope C1/C2** | 24 (48) | 52 (47.27) | Ref | | 41 (47.67) | 9 (37.5) | 2.02 (0.6-6.77) | 0.2525 | 39 (52) | 2 (18.18) | Ref | |
| **Epitope C1/C1** | 15 (30) | 28 (25.45) | 0.91 (0.34-2.42) | 0.851 | 21 (24.42) | 10 (41.67) | Ref | | 15 (20) | 6 (54.55) | 75.1 (1.48-3822.56) | **0.031** |
| **Epitope C2/C2** | 11 (22) | 30 (27.27) | 1.18 (0.42-3.3) | 0.750 | 24 (27.91) | 5 (20.83) | 3.38 (0.82-14) | 0.093 | 21 (28) | 3 (27.27) | 44.63 (1.16-1716.2) | **0.041** |

^a^Odds ratios were adjusted by gender, skin color, education, site of tuberculosis, HIV status, CD4 count, and CD4/CD8 ratio when appropriate. ^b^P-values were calculated using the unconditional logistic regression model. ^c^The HLA-C determination was not possible for two individuals from G1. So, when considering this variable, N (G1) = 86 and N (G1+G3) = 110. Differences were considered significant with a value of * P < 0.05. N: number of individuals in each group; OR: odds ratio; aOR: adjusted odds ratio; 95% CI: 95% confidence interval; REF: Reference; IRIS: immune reconstitution inflammatory syndrome; NC: not calculated.

Table S4: Distribution of HLA-B alleles of the subjects included in this study and the Brazilian general population (data from the Brazilian Registry of Bone Marrow Donors - REDOME).

| **HLA-B alleles** | **REDOME** | | **All the groups** | | **REDOME vs all the groups** |
| --- | --- | --- | --- | --- | --- |
|  | **2N= 5,695,736** | | **2N=324** | |  |
|  | **n** | **Freq** | **n** | **Freq** | **P-value^a^** |
| **B*07** | 393,814 | 6.90% | 23 | 7.10% | 1.000 |
| **B*08** | 291,107 | 5.10% | 16 | 4.90% | 1.000 |
| **B*13** | 90,593 | 1.60% | 5 | 1.50% | 0.988 |
| **B*14** | 300,037 | 5.30% | 18 | 5.60% | 0.994 |
| **B*15** | 518,155 | 9.10% | 43 | 13.30% | **0.042** |
| **B*18** | 270,883 | 4.80% | 9 | 2.80% | 0.304 |
| **B*21** | 0 | 0% | 1 | 0.30% | NC |
| **B*27** | 126,605 | 2.20% | 3 | 0.90% | 0.378 |
| **B*35** | 673,880 | 11.80% | 30 | 9.30% | 0.403 |
| **B*37** | 60,869 | 1.10% | 2 | 0.60% | 0.874 |
| **B*38** | 121,887 | 2.10% | 8 | 2.50% | 0.977 |
| **B*39** | 197,423 | 3.50% | 8 | 2.50% | 0.709 |
| **B*40** | 273,605 | 4.80% | 8 | 2.50% | 0.186 |
| **B*41** | 73,500 | 1.30% | 5 | 1.50% | 0.988 |
| **B*42** | 79,525 | 1.40% | 9 | 2.80% | 0.170 |
| **B*44** | 615,800 | 10.80% | 33 | 10.20% | 0.963 |
| **B*45** | 98,905 | 1.70% | 9 | 2.80% | 0.474 |
| **B*46** | 2,365 | 0% | 0 | 0% | 0.609 |
| **B*47** | 12,556 | 0.20% | 0 | 0% | 0.968 |
| **B*48** | 41,436 | 0.70% | 4 | 1.20% | 0.756 |
| **B*49** | 158,232 | 2.80% | 6 | 1.90% | 0.700 |
| **B*50** | 136,173 | 2.40% | 4 | 1.20% | 0.498 |
| **B*51** | 475,056 | 8.30% | 16 | 4.90% | 0.107 |
| **B*52** | 110,700 | 1.90% | 8 | 2.50% | 0.889 |
| **B*53** | 135,283 | 2.40% | 19 | 5.90% | **<0.001** |
| **B*54** | 3,670 | 0.10% | 0 | 0% | 0.816 |
| **B*55** | 62,186 | 1.10% | 1 | 0.30% | 0.553 |
| **B*56** | 20,859 | 0.40% | 1 | 0.30% | 0.959 |
| **B*57** | 159,435 | 2.80% | 11 | 3.40% | 0.890 |
| **B*58** | 151,176 | 2.70% | 16 | 4.90% | 0.058 |
| **B*59** | 973 | 0% | 0 | 0% | 0.168 |
| **B*67** | 1,579 | 0% | 0 | 0% | 0.392 |
| **B*73** | 5,648 | 0.10% | 0 | 0% | 0.951 |
| **B*78** | 4,956 | 0.10% | 1 | 0.30% | 0.919 |
| **B*81** | 24,196 | 0.40% | 6 | 1.90% | **0.002** |
| **B*82** | 2,658 | 0% | 1 | 0.30% | 0.669 |

^a^P-values were calculated using the unconditional logistic regression model. Differences were considered significant with a value of * P < 0.05. REDOME= Brazilian National Registry of Bone Marrow Donors; N: number of individuals in each group; n: observed number of an allele/genetic marker carriers; Freq: relative frequency of carriers for each allele/genetic marker, calculated as the number of carriers/2N; NC: not calculated.

Table S5: Frequency of KIR genotypes and mapping of the inhibitory/activator genes among the subjects included in this study.

Subtitle: Black box (presence) and white box (absence).

Table S6: Distribution of KIR genotypes of subjects included in this study stratified by groups.

| KIR Genotypes | All the groups | | | | HIV-1 positive individuals | | | | IRIS | | | |
| --- | --- | --- | --- | --- | --- | --- | --- | --- | --- | --- | --- | --- |
|  | **With TB (G1+G3)** | **Without TB (G2+G4)** | **aOR**  **(95% CI)** | **p-valor** | **With TB (G1)** | **Without TB (G2)** | **aOR**  **(95% CI)** | **p-valor** | **Without IRIS** | **With IRIS** | **aOR**  **(95% CI)** | **p-valor** |
|  | **N=50** | **N=112** |  |  | **N=88** | **N=24** |  |  | **N=77** | **N=11** |  |  |
| **Genotypes Bx** | 32 (64) | 80 (71.46) | Ref | Ref | 62 (70.45) | 16 (66.67) | Ref | Ref | 52 (67.53) | 10 (90.91) | Ref | Ref |
| **Genotypes AA** | 18 (36) | 32 (28.57) | 0.44 (0.18-1.12) | 0.084 | 26 (29.55) | 8 (33.33) | 0.62 (0.22-1.78) | 0.380 | 25 (32.41) | 1 (9.09) | 0 (0-Inf) | 0.995 |

^a^Odds ratios were adjusted by gender, skin color, education, site of tuberculosis, HIV status, CD4 count, and CD4/CD8 ratio when appropriate. ^b^P-values were calculated using the unconditional logistic regression model. Differences were considered significant with a value of * P < 0.05. N: number of individuals in each group; OR: odds ratio; aOR: adjusted odds ratio; 95% CI: 95% confidence interval; REF: Reference; IRIS: immune reconstitution inflammatory syndrome.

Table S7: Sociodemographic, clinical, and laboratory data of HIV-TB individuals with and without IRIS.

| **Features** | **Overall** | **Without IRIS**  **N=77** | **With IRIS**  **N=11** | ***P*-value^a^** |
| --- | --- | --- | --- | --- |
| **Gender; n (%)** | | | | |
| **Female** | 18 (20.5) | 18 (20.5) | 0 (0) | 0.111 |
| **Male** | 70 (79.5) | 59 (67) | 11 (12.5) |  |
| **Skin Color; n (%)** | | | | |
| **Black** | 21 (23.9) | 18 (20.5) | 3 (3.4) | 0.389 |
| **Brown** | 32 (36.4) | 30 (34.1) | 2 (2.3) |  |
| **White** | 35 (39.8) | 29 (33) | 6 (6.8) |  |
| **Education^b^; n (%)** | | | | |
| **Bachelor** | 3 (3.4) | 2 (2.3) | 1 (1.1) | 0.313 |
| **Upper-secondary** | 19 (21.6) | 17 (19.3) | 2 (2.3) |  |
| **Lower-secondary** | 38 (43.2) | 31 (35.2) | 7 (8) |  |
| **Primary** | 23 (26.1) | 22 (25) | 1 (1.1) |  |
| **Unknown** | 5 (5.7) | 5 (5.7) | 0 (0) |  |
| **Site Of Tuberculosis; n (%)** | | | | |
| **Pulmonary** | 46 (52.3) | 43 (48.9) | 3 (3.4) | 0.108 |
| **Disseminated/Extrapulmonary** | 42 (47.7) | 34 (38.6) | 8 (9.1) |  |
| **HIV transmission route; n (%)** | | | | |
| **Heterosexual/Oth** | 66 (75) | 61 (69.3) | 5 (5.7) | **0.027** |
| **MSM** | 20 (22.7) | 15 (17) | 5 (5.7) |  |
| **Unknown** | 2 (2.3) | 1 (1.1) | 1 (1.1) |  |
| **CD4 count (cell/µL) (IQR)** | 89 (143.7) | 92.5 (153.5) | 50 (68.5) | 0.123 |
| **(≤50); n (%)** | 28 (33.3) | 23 (26.1) | 5 (5.7) | 0.289 |
| **(>50); n (%)** | 56 (66.7) | 51 (58) | 5 (5.7) |  |
| **CD8 count (IQR)** | 599 (634.5) | 628 (591) | 424.5 (875.2) | 0.545 |
| **(≤500); n (%)** | 29 (36.7) | 22 (25) | 7 (8) | **0.032** |
| **(>500); n (%)** | 50 (63.3) | 47 (53.4) | 3 (3.4) |  |
| **CD4/CD8 ratio (IQR)** | 0.13 (0.29) | 0.13 (0.29) | 0.08 (0.1) | 0.221 |
| **(≤0.15); n (%)** | 46 (58.2) | 39 (44.3) | 7 (8) | 0.508 |
| **(>0.15); n (%)** | 33 (41.8) | 30 (34.1) | 3 (3.4) |  |
| **Viral load (copies/mL) (IQR)** | 221,184 (444,669) | 195,969 (417,602) | 281,629 (962,631) | 0.250 |
| **(<20000); n (%)** | 39 (48.8) | 35 (39.8) | 4 (4.5) | 0.738 |
| **(>20000); n (%)** | 41 (51.2) | 35 (39.8) | 6 (6.8) |  |
| **LogCV (IQR)** | 5.34 (0.96) | 5.29 (1.01) | 5.44 (1.14) | 0.250 |

^a^P-values were calculated using Fisher’s exact test. Differences were considered significant with a value of * P < 0.05. ^b^Classification, according to the International Standard Classification of Education (ISCED) maintained by the United Nations Educational, Scientific and Cultural Organization (UNESCO). N: number of individuals in each group; IQR: interquartile range; VL: viral load; IRIS: immune reconstitution inflammatory syndrome.
